# Exploring adolescent suicide attempts before and during the coronavirus disease 2019 pandemic: a cross-sectional study

**DOI:** 10.1590/1984-0462/2025/43/2024093

**Published:** 2024-11-29

**Authors:** Lucca Garcia Moreira Ribeiro, Danton Matheus de Souza, Carlos Alberto dos Santos Treichel, Vanessa Dias Fogaça, Lisabelle Mariano Rossato

**Affiliations:** a Universidade de São Paulo, Escola de Enfermagem, São Paulo, SP, Brazil.; b Universidade de São Paulo, Hospital Universitário, SP, Brazil.

**Keywords:** Adolescent, Suicide, attempted, COVID-19, Adolescent health, Mental health, Adolescente, Tentativa de suicídio, COVID-19, Saúde do adolescente, Saúde mental

## Abstract

**Objective:**

The objective of this study was to analyze suicide attempts in adolescents treated in the emergency department before and during the coronavirus disease 2019 pandemic.

**Methods:**

This cross-sectional, documentary, and retrospective study assessed the medical records of adolescents treated for suicide attempts in two emergency rooms linked to a teaching hospital in São Paulo, Brazil. Data were collected from the pre-pandemic period (between January 1, 2015, and March 10, 2020) and the pandemic period (between March 11, 2020, and May 5, 2023), characterizing the adolescents, risk factors for suicide attempts during the pandemic, and treatment. Data were subjected to descriptive and inferential analyses, including association tests and trend analysis, with a p-value of <0.05 considered statistically significant. The national ethical guidelines were followed.

**Results:**

Data on 140 suicide attempts were collected, of which 54 occurred during the pandemic. The trend in these cases remained stable (p>0.05). We observed an association between adolescents who had completed high school and cases during the pandemic, with a previous mental disorder diagnosis and pre-pandemic cases, and a higher number of suicide attempt notifications to the Brazilian epidemiological surveillance system during the pandemic period (p<0.05). Feelings of loneliness were the most prevalent risk factor during the pandemic.

**Conclusions:**

The COVID-19 pandemic did not show an influence on the number of suicide attempt cases in adolescents. However, it was observed that the cases during this period were not related to previous mental disorder diagnoses, as seen in the pre-pandemic period, which may suggest that other risk factors were associated with suicide attempt cases during the pandemic.

## RESUMO

**Objetivo:**

Analisar as tentativas de suicídio em adolescentes atendidos em um departamento de urgência e emergência antes da pandemia da COVID-19 e durante seu curso.

**Métodos:**

Estudo transversal, documental e retrospectivo, conduzido com prontuários de adolescentes atendidos por tentativa de suicídio, em dois prontos-socorros vinculados a um hospital escola no município de São Paulo, Brasil. Foram coletados dados do período pré-pandêmico (entre 1^o^ de janeiro de 2015 e 10 de março de 2020) e período pandêmico (entre 11 de março de 2020 e 5 de maio de 2023), com a caracterização do adolescente, dos fatores de risco à tentativa de suicídio na pandemia e do atendimento. Os dados foram submetidos a análise descritiva e inferencial, com testes de associação e análise de tendência, considerando-se o valor de p<0,05 como diferença estatística. Respeitaram-se as diretrizes éticas nacionais.

**Resultados:**

Foram coletados dados de 140 atendimentos de tentativa de suicídio, dos quais 54 ocorreram no período pandêmico. A tendência de atendimentos mostrou-se estacionária (p>0,05). Observou-se associação entre os adolescentes que estudaram até o ensino médio e os atendimentos na pandemia; ter um diagnóstico de transtorno mental prévio e o atendimento pré-pandêmico; e o maior número de notificações de tentativa de suicídio para o sistema de vigilância epidemiológico brasileiro no período pandêmico (p<0,05). A sensação de solidão destacou-se como fator de risco pandêmico mais prevalente.

**Conclusões:**

A pandemia da COVID-19 não mostrou influência nos atendimentos de tentativa de suicídio em adolescentes. Todavia, observou-se que os casos neste período não tiveram relação com o diagnóstico de transtorno mental prévio, como visto no período pré-pandêmico, o que pode sugerir que outros fatores de risco estiveram associados aos casos de tentativa de suicídio durante a pandemia.

## INTRODUCTION

Toward the end of 2019, the coronavirus disease 2019 (COVID-19) emerged as responsible for an increase in cases of acute respiratory syndromes in China. The virus quickly spread worldwide, resulting in the declaration of a global pandemic by the World Health Organization (WHO) in March 2020.^1^ When comparing adolescents with the adult population, it is observed that the proportion of COVID-19 cases is smaller in this public. The clinical implications of the infection involve less severe symptoms and a better prognosis.^2^ Nonetheless, the pandemic has led to significant impacts on the mental health of youth, with an increase in symptoms of mental disorders and rates of self-inflicted violence.^3,4^ The combination of the COVID-19 pandemic with mental health demands has resulted in a syndemic, a concept defined as a process of synergistic interaction between two or more health issues that amplify their repercussions.^5^ In this context, special attention should be given to suicidal behavior during the COVID-19 pandemic.

Suicidal behavior can be defined as a series of acts that include ideation, planning, attempts, and suicide. Suicidal ideation is related to the desire to end one’s own life without any resulting action and may be accompanied by planning for future actions. Suicide attempts, the focus of this study, are characterized by any nonfatal suicidal behavior, such as exogenous intoxication and self-inflicted injuries that are associated with ending one’s life. Suicide is defined as the deliberate act of taking one’s own life.^6^

Globally, suicide is the second leading cause of death among young people aged 10–24 years, with an estimated three suicides per 100,000 adolescents.^7,8^ Over the past 10 years, in Brazil, suicides of individuals aged 10–19 years accounted for 8.5% of all suicides and 4.4% of all deaths within this age group.^9^ However, the magnitude of these data may be underestimated due to underreporting and misclassification of suicides as deaths from external causes. Regarding suicide attempts, it is estimated that they outnumber suicides by 10 times.^10^ A Brazilian population-based survey found a lifetime prevalence of 2.8% for suicide attempts.^11^ Meanwhile, a meta-analysis involving a population of 686,672 children and adolescents found a prevalence of 6% for suicide attempts.^12^ Notably, one-third of all suicide attempts are initially treated in emergency departments, highlighting the need to pay attention to these services.^11^

A systematic review of data from low- and middle-income countries found heterogeneous results regarding the prevalence of suicidal behaviors across different regions of the world, with high suicide rates among adolescents in low-income countries compared to those in high-income countries. However, the article highlights the scarcity of studies in low- and middle-income countries, such as Brazil, especially regarding the influence of the COVID-19 pandemic on the phenomenon.^13^ Thus, this study aimed to analyze suicide attempts by adolescents treated in an emergency department before and during the COVID-19 pandemic.

## METHOD

A cross-sectional study was conducted using the medical records of adolescents treated for suicide attempts in the emergency department of a public teaching hospital located in São Paulo, Brazil. It provides care to the population dependent on the Brazilian Unified Health System (SUS) and serves residents in the western region of the state, especially those from lower economic classes. There are two emergency services: one is for children providing care for adolescents between 10 and 15 years old, and the other is for adults attending to adolescents between 15 and 19 years of age.

The inclusion criteria were as follows: medical records of adolescent patients aged between 10 and 19 years, as defined by the WHO,^14^ for suicide attempts between January 2015 and May 2023. To identify medical records, the International Statistical Classification of Diseases and Related Health Problems, 10th edition (ICD-10)^15^ was used with the following codes: X60 to X84 (suicide attempt), T36 to T78 (poisoning), X60 to X84 (self-inflicted injuries), T50.9 to T65.9 (exogenous intoxication of undetermined intent), and Y06 to X34 (accident of undetermined intent). No exclusion criteria were established.

Notably, the data collection timeframe was defined to capture the medical records of adolescents treated in the pre-pandemic period (between January 1, 2015, and March 10, 2020) and the pandemic period (between March 11, 2020, and May 5, 2023), according to WHO statements.^1,16^ Considering that a previous study^17^ demonstrated a low number of suicide attempt notifications, it was decided to track medical records by ICD-10 codes beyond suicide attempts themselves, with a focus on the means used for the attempt. Additionally, no prior sample size was calculated, and data were collected from all eligible medical records within the established period.

For data collection, medical and statistical archive services from the participating institutions were requested to provide a list of medical records that met the defined criteria. A total of 420 medical records were collected and evaluated. Medical records were classified as suicide attempts if there was documentation of intentional action, implicitly or explicitly evidenced, indicating the desire to take one’s own life.^17^ Among them, 140 were characterized as suicide attempts.

The analysis of medical records and data extraction were performed by a qualified researcher: a nurse specializing in child and adolescent health with extensive experience in treating adolescent suicide attempts. An instrument was formulated for data collection with objective questions and closed- or open-ended responses. Data were collected regarding the characterization of the adolescent: age, sex, race (classified as White and non-White — mixed race, Asian, Indigenous, and Black), education level, mental disorders (for those with at least one previous psychiatric diagnosis, as coded in the medical records as part of the ICD-10; examples include depressive episode disorders, anxiety disorders, personality and behavior disorders, among others), and use of any continuous psychotropic medication. Characterization of risk factors for suicide attempts during the pandemic was categorized into emotional, family, economic, and social stressors, as well as factors related to mental health. The authors used previous studies that focused on this aspect to identify risk factors and their classification.^18-23^ Data related to suicide attempt treatment included location, method used, transportation to the service, day of treatment, resources used for treatment, discharge conduct (no conduct, referral not addressed to the network, and referral addressed to the network), and completion of the self-inflicted violence notification form.

The data were tabulated and subjected to descriptive and inferential analysis using Stata18^®^ software (Stata Corporation, College Station, Texas, USA). For descriptive analysis, percentiles, measures of central tendency (mean), and dispersion (standard deviation) were used. For inferential analysis, after testing the nature of the variable distribution, Pearson’s chi-square test was used to verify the existence of an association between dependent variables (treatment conducted during the pandemic) and independent variables. For trend analysis, a Poisson regression model was used, assuming significant changes in suicide attempt treatments during the pandemic period when the p-value was less than 0.05 (5%). Odds ratios (OR) were used to identify the probability of changes over time. The precision of the OR was measured using 95% confidence intervals (95% CI). Statistical significance was set at p<0.05 (5%) and was considered statistically significant — 95% confidence interval.

The study was approved by the Nursing School of the University of São Paulo and participating institutions. A waiver of consent was granted, with the principal researcher signing the Responsibility Term. The Strengthening the Reporting of Observational Studies in Epidemiology (STROBE) guidelines for cross-sectional studies were used for data reporting.

## RESULTS

Data on 140 suicide attempts were collected during the established period. There was a predominance of adolescents aged 15–19 years. Further, most were White women who had completed Brazilian high school education, with suicide attempts during the pandemic period being more prevalent among adolescents who had achieved this level of education (p<0.001). Moreover, 67 (47.8%) adolescents had at least one previous diagnosis of a mental disorder, which was more frequent among those who attempted suicide in the pre-pandemic period (p<0.001). Furthermore, 89 (63.6%) were using at least one continuous psychotropic medication. The other variables showed no association when comparing the pre-pandemic and pandemic periods. Table 1 presents the characteristics of the participants and the associations between the periods.

**Table 1:** Characterization of treated adolescents and their association with the pandemic period in São Paulo (SP), Brazil; 2023.

	Pre-pandemic period (n=86)	Pandemic period (n= 54)	p-value
	n (%)	n (%)	
Age (years)			
Incomplete 10–14	22 (25.6)	13 (23.1)	0.841
Incomplete 15–19	64 (74.4)	41 (75.9)	
Sex			
Female	71 (82.6)	42 (77.8)	0.485
Male	15 (17.4)	12 (22.2)	
Race			
White	71 (82.6)	42 (77.8)	0.485
Non-White	15 (17.4)	12 (22.2)	
Education level			
Up to elementary school	38 (44.2)	6 (11.1)	**<0.001**
Up to high school	37 (43.0)	39 (72.2)	
Up to higher education	11 (12.8)	9 (16.7)	
Previous mental disorders			
Yes	51 (59.3)	16 (29.6)	**<0.001**
No	35 (40.7)	38 (70.4)	
Current use of psychotropic medication			
Yes	60 (69.8)	29 (53.7)	0.055
No	26 (30.2)	25 (40.3)	

Statistically significant values are denoted in bold.

**Figure 1:**
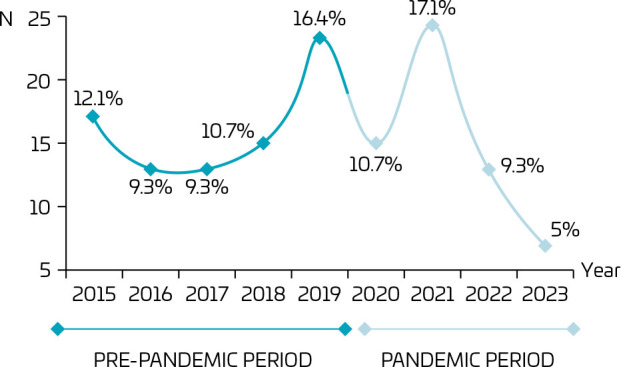
Trend of cases of suicide attempts over the years (2015–2023) in São Paulo (SP), Brazil; 2023.

The treatments maintained a stable trend over the years (OR 1.002; 95%CI 0.99–1.00; p=0.482), without any influence from the pandemic period. Figure 1 shows the trend of cases over the years, in the pre-pandemic and pandemic periods.

Fifty-four adolescents were treated during the pandemic, and numerous risk factors were identified. The following factors prevailed during the pandemic: feelings of loneliness, a diagnosis of mental disorders, and increased family conflict. Table 2 shows the risk factors along with their prevalence rates.

**Table 2:** Characterization of risk factors for suicide attempts during the pandemic (n=54) in São Paulo (SP), Brazil; 2023.

Variables*	n	Prevalence (%)
Emotional stress factors		
The feeling of loneliness in the pandemic	39	72.2
Change of residence in the pandemic	6	11.1
Fear related to COVID-19 infection	3	5.6
Diagnosis of COVID-19	3	5.6
Low academic performance during the pandemic	1	1.8
Grief due to loss of a loved one to COVID-19	1	1.8
Family stress factors		
Increased family conflicts during the pandemic	29	53.7
Economic stress factors		
Financial difficulties during the pandemic	14	25.9
Reduction in family income during the pandemic	6	11.1
Job loss by family members during the pandemic	5	9.3
Social stress factors		
Increased social conflicts during the pandemic	19	35.2
Social isolation	13	24.1
Factors associated with mental health		
Diagnosed mental disorder during the pandemic	31	57.4
Initiation of psychotropic medication during the pandemic	22	40.7
Initiation of psychological counseling during the pandemic	16	29.6
Discontinuation of psychological counseling during the pandemic	8	14.8
Discontinuation of psychotropic medication use during the pandemic	6	11.1

*Each adolescent could have more than one risk factor.

Suicide attempts at home were prevalent through intoxication, with transportation to emergency rooms mainly by family members on weekdays. Psychiatric professionals predominantly use psychiatric and social service assessments as treatment resources. Regarding discharge, the most common method was referring an adolescent to a Psychosocial Care Network (RAPS) facility. It is noteworthy that during the pandemic period, completion of the self-inflicted violence notification form was more frequent (p=0.004). The other variables were not associated with the pandemic period (Table 3).

**Table 3:** Treatment of suicide attempts, outcomes, and associations with the pandemic period in São Paulo (SP), Brazil; 2023.

Variables	Pre-pandemic period (n=86)	Pandemic period (n=54)	p-value
	n (%)	n (%)	
Location of suicide attempt			
Residence	80 (93.0)	49 (90.7)	0.625
External	6 (7.0)	5 (9.3)	
Means used for the suicide attempt			
Pharmacological intoxication	74 (86.0)	43 (79.6)	0.319
Poisoning	8 (9.3)	5 (9.3)	0.993
Alcohol or drug intoxication	7 (8.1)	5 (9.3)	0.818
Stab wound	2 (2.3)	5 (9.3)	0.067
Falling from height	2 (2.3)	1 (1.8)	0.851
Hanging	0 (0)	1 (1.8)	0.205
Trauma	0 (0)	1 (1.8)	0.205
Transport to the emergency room			
Family	63 (73.2)	39 (72.2)	0.097
Healthcare network	1 (1.2)	5 (9.3)	
Self-referral	18 (20.9)	7 (12.9)	
Others	4 (4.7)	3 (5.6)	
Day of treatment			
Weekdays	74 (86.1)	47 (87.0)	0.868
Weekend	12 (13.9)	7 (13.0)	
Resources used for the treatment*			
Psychiatrist assessment	58 (68.6)	34 (62.9)	0.491
Social service assessment	30 (34.8)	18 (33.3)	0.851
Emergency room	29 (33.7)	15 (27.8)	0.461
Ward admission	13 (15.1)	9 (16.6)	0.806
Intensive care unit admission	7 (8.1)	7 (12.9)	0.354
Discharge procedures			
Discharge without procedures	39 (68.4)	18 (31.6)	0.159
Referral addressed to the network	26 (61.9)	16 (38.1)	0.940
The referral was not addressed to the network	21 (51.2)	20 (48.8)	0.110
Notification of suicide attempt			
Yes	17 (19.7)	23 (42.6)	**0.004**
No	69 (80.2)	31 (57.4)	

*Each adolescent may have used multiple methods and resources during treatment.

Statistically significant value is denoted in bold.

## DISCUSSION

In this study, although the pandemic period did not show an influence on the trend of suicide attempt cases in adolescents in the emergency department, it is noteworthy that the attempts during this period occurred in adolescents without a history of mental disorders, which may indicate the pandemic’s influence on other risk factors. In comparison with the pre-pandemic period, there was an observed association between suicide attempts and the diagnosis of mental disorders. Additionally, this study revealed a low number of reported suicide attempt cases, although there was an increase in notifications to the Brazilian epidemiological surveillance system during the pandemic period.

In the literature, there is still divergence in the trend of suicide attempts during the pandemic. Some studies indicate an increase in cases,^19,20^ while others suggest a reduction during the period.^24^ A cross-sectional research conducted in Italy with mental health care from January 2011 to May 2022 showed an increase in suicide attempt cases over the years, with particular attention to the rise after the declaration of the COVID-19 pandemic.^19^ Contrastingly, a cross-sectional study in Israel indicated a decrease in the number of suicide attempt cases in children and adolescents during the pandemic, especially in the first wave of COVID-19.^24^ This study observed a stationary trend in the number of cases, suggesting no influence from the pandemic.

The literature hypothesizes that initially, shortly after the pandemic declaration, cases of suicide attempts decreased, while after the loosening of social distancing measures, cases increased to a level close to the pre-pandemic.^21,24^ At the beginning of the pandemic, with lockdown measures in place, teenagers stayed at home under family supervision, while health services were reorganized with a focus on acute COVID-19 cases. In this scenario, there was a possibility that many mental health demands and suicide attempts did not reach these services, considering the population’s fear of contracting the virus when seeking help.^21,24^ After the first wave of cases and subsequent gradual return to activities, especially in 2021, studies indicate an increase in the number of suicide attempts, which may be associated with a change in the abovementioned context and a return to exposure to social risk factors.^19,20^ Along this path, it is essential to pay attention to factors that may be related to the risk or protection against suicide attempts.^17^

Regarding mental health risk factors, there has been an increase in the number of children and adolescents diagnosed with mental health disorders during the pandemic.^20,23^ There is an indication that having a disorder increases the chance of suicidal behavior by three to twelve times.^25^ It is important to note that this study observed a discrepancy between the number of adolescents with a clinical diagnosis of a mental health disorder and the use of psychotropic medications. This aspect may prompt reflection on the social representation of psychotropic medications as the primary form of care for mental health needs, even when these are not associated with a clinical diagnosis. This context needs to be further explored in future studies.

This study highlights the prevalence of emotional risk factors, such as loneliness, during the pandemic, affecting 72.2% of adolescents. Confinement reduced peer interactions, which may have been detrimental to adolescents, who are highly dependent on relationships, which are essential for psychosocial development and are a significant source of emotional support.^20^ Other emotional influences on adolescent mental health, as indicated in a systematic review, include worry, helplessness, fear, nervousness, agitation, and aggression.^23^

A factor that can be both protective and risky for adolescent suicide attempts is their relationship with their schools. In this study, an association was observed between suicide attempts during the pandemic period and high school students (p<0.001). This aspect may be related to the social pressure of career choice that affects high school adolescents, which may have increased during the pandemic, especially due to the need for entrance exams. At the same time, the interruption of academic activities may have reduced adolescents’ prospects for their professional future. Concurrently, attending school can protect adolescents by reducing exposure to other risk factors, such as family conflicts.

Family is fundamental to establishing emotional bonds and strengthening adolescents’ psychosocial well-being. However, the family can also be a source of struggle.^26^ In a cross-sectional study conducted in Poland with 154 adolescents who attempted suicide, 13.6% indicated family as a potential source of strain, primarily due to conflicts at home.^27^ These conflicts may have intensified during the pandemic, particularly during social isolation, with increased cohabitation, supporting the finding of this study that identified family conflicts as a risk factor for suicide attempts.^13,26^ In this context, family inclusion in care is vital, such as through family interventions that reframe interaction from draining to empowering.^25^

Economic hardship is a risk factor for mental health problems, as seen in a North American study that found an association between job loss among family members during the pandemic period and suicidal behavior in high school adolescents.^28^ During this period, difficulties may have been exacerbated by the impending economic crisis, as seen in this study, corroborating another Brazilian investigation conducted with 51 families of children and adolescents with COVID-19, indicating a prevalence of 78.4% of economic impacts during the pandemic, characterized as significant by almost half of them, and being higher in those with lower income and those dependent on social benefits.^26^

In the characterization of suicide attempt cases, the only variable that showed a statistically significant difference in this study was notification to the Brazilian epidemiological surveillance system. In Brazil, notification of self-inflicted violence cases is compulsory and must be recorded within 24 h of the event becoming known. It plays a unique role in case management by initiating coordination within the network.^17^ This study draws attention to the association between increased notification numbers during the pandemic, possibly due to the higher visibility that health surveillance services received during this period for their role in estimating COVID-19 cases. Furthermore, the increased attention given to mental health outcomes may have sensitized healthcare professionals to make greater use of notification forms. Nonetheless, the reported cases remain low, accounting for half of the total attempts.

The absence of association with other variables such as method, location, and resources corroborates the literature. This indicates that cases did not worsen during the pandemic, which was predominantly characterized by exogenous intoxications occurring at home, transportation to healthcare services by family members, and clinical stabilization treatment.^20,25^

Despite the discharge practices not being associated with the pandemic, the high number of referrals not directed to the network in both periods is noteworthy. In such cases, adolescents and their families are expected to autonomously seek subsequent care at a healthcare facility, which may lead to a more significant discontinuity in care than directed referrals. However, the literature estimates that regardless of the period, more than 50% of adolescents with mental health problems are not served within the network.^29^ In contrast, during the pandemic period, the possibility of care may have diminished as healthcare services reorganized to handle the high demand caused by acute COVID-19 cases.^19^ The literature recommends an implied and co-responsible referral, with addressing and coordination between the referring and receiving services.^30^ Efforts to ensure these recommendations are necessary.

This study has some limitations. It was conducted in only one health service with a retrospective design that does not allow causal inference. Further, it relies on the quality of the medical record documentation and may be limited by underreported suicide attempts, which require data collection under different criteria. It is hoped that this will contribute to the reflection of healthcare professionals and the development of strategies to mitigate the effects of the pandemic on adolescent health, especially concerning suicidal behavior. The innovative nature of this study is emphasized, as previous studies have focused on pandemic snapshots, whereas this study encompassed the entire pandemic period.

In this study, the pandemic period did not influence adolescent suicide attempt admissions, and the trend remained stable. Only the association between adolescents who studied up to high school and admissions during the pandemic was observed — notably, with a previous diagnosis of mental disorder and pre-pandemic admissions and a higher number of suicide attempt notifications during the pandemic period. Furthermore, during the pandemic period, despite the absence of an association, it is noteworthy that the majority of cases occurred in adolescents without a history of mental disorders, which may indicate that other risk factors were linked to suicidal behavior, such as loneliness.

This study highlights the importance of reflecting on future strategies for promoting mental health and minimizing the potential long-term consequences of the pandemic period. Additionally, increasing the number of reported cases is crucial to give visibility to the issue and to encourage the translation of current public policies into clinical practice.

## Data Availability

The database that originated the article is available with the corresponding author.
